# Utility of broad-spectrum antibiotics for diagnosing pulmonary tuberculosis in adults: a systematic review and meta-analysis

**DOI:** 10.1016/S1473-3099(20)30143-2

**Published:** 2020-09

**Authors:** Titus H Divala, Katherine L Fielding, Chikondi Kandulu, Marriott Nliwasa, Derek J Sloan, Ankur Gupta-Wright, Elizabeth L Corbett

**Affiliations:** a TB Centre, London School of Hygiene & Tropical Medicine, London, UK; b Helse Nord Tuberculosis Initiative, University of Malawi College of Medicine, Blantyre, Malawi; c School of Public Health, University of the Witwatersrand, Johannesburg, South Africa; d School of Medicine, University of St Andrews, St Andrews, UK; e Malawi Liverpool Wellcome Trust Clinical Research Programme, Blantyre, Malawi

## Abstract

**Background:**

Suboptimal diagnostics for pulmonary tuberculosis drive the use of the so-called trial of antibiotics, a course of broad-spectrum antibiotics without activity against *Mycobacterium tuberculosis* that is given to patients who are mycobacteriology negative but symptomatic, with the aim of distinguishing pulmonary tuberculosis from bacterial lower respiratory tract infection. The underlying assumption—that patients with lower respiratory tract infection will improve, whereas those with pulmonary tuberculosis will not—has an unclear evidence base for such a widely used intervention (at least 26·5 million courses are prescribed per year). We aimed to collate available evidence on the diagnostic performance of the trial of antibiotics.

**Methods:**

In this systematic review and meta-analysis we searched the MEDLINE, Embase, and Global Health databases for studies published up to March 15, 2019, that investigated the sensitivity and specificity of the trial of antibiotics against mycobacteriology tests in adults (≥15 years) with tuberculosis symptoms. We used the QUADAS-2 tool to assess the risk of bias. We estimated pooled values for sensitivity and specificity of trial of antibiotics (as the index text) versus mycobacteriology tests (as the reference standard) using random-effects bivariate modelling, and we used the *I*^2^ statistic to assess heterogeneity between studies contributing to these estimates. This study is registered with PROSPERO, number CRD42017083915.

**Findings:**

Of the 9410 articles identified by our search, eight studies were eligible for inclusion. The studies were from seven countries in Africa, South America, and Asia, and involved 2786 participants. Six studies used mycobacterial culture as the reference standard, and six used penicillins for the trial of antibiotics. The treatment duration, number of antimicrobial courses, and definition of what constituted response to treatment varied substantially between studies. The pooled sensitivity (67%, 95% CI 42–85) and specificity (73%, 58–85) of the trial of antibiotics versus mycobacteriology tests were below internationally defined minimum performance profiles for tuberculosis diagnostics and had substantial heterogeneity (*I*^2^ was 96% for sensitivity and 99% for specificity). Each included study failed on one or more domain of the QUADAS-2 tool.

**Interpretation:**

Current policy and practice regarding the trial of antibiotics appear inappropriate, given the weak evidence base, poor diagnostic performance, potential contribution to the global antimicrobial resistance crisis, and adverse individual and public health consequences from the misclassification of tuberculosis status. Antibiotic strategies during tuberculosis investigations should instead optimise clinical outcomes, ideally guided by clinical trials in both inpatient and outpatient groups.

**Funding:**

Helse Nord RHF, Wellcome Trust, and the UK Commonwealth Scholarship Commission.

## Introduction

Tuberculosis is the leading cause of adult mortality due to infectious disease, with 10 million new cases and 1·6 million deaths annually,^1^ but it is curable when correctly diagnosed in a timely manner. However, current diagnostics are suboptimal, missing many cases.^2^ Recognising the limitations of current diagnostic tests, the standard diagnostic algorithms that are endorsed by WHO^3^, ^4^ and that have been routinely promoted by national tuberculosis programmes^5^ include the level of response to a course of broad-spectrum antibiotics as a means of excluding (or including) tuberculosis as a cause of symptoms. The course of broad-spectrum antibiotics, commonly referred to as a trial of antibiotics, has negligible activity against *Mycobacterium tuberculosis* (MTB) and is given to symptomatic patients with negative sputum mycobacteriology (panel, appendix pp 3–4).^6^ Patients with negative sputum mycobacteriology whose symptoms respond to the antibiotic treatment are considered tuberculosis negative, whereas those who remain symptomatic are deemed in need of further evaluations, potentially leading to tuberculosis treatment.^6^, ^7^

Panel **Antibiotics as diagnostics for tuberculosis**

Tuberculosis should be investigated in all patients presenting with respiratory symptoms using sputum-based tuberculosis diagnostic tests (smear microscopy or Xpert MTB/Rif). However, negative results on these tests do not rule out tuberculosis. The 2018 WHO model diagnostic algorithm (appendix p 3) advises clinical re-evaluation of patients with negative sputum results, with suggestions of “chest X-ray, additional clinical assessments, clinical response following treatment with broad-spectrum antimicrobial agents, repeat Xpert MTB/RIF testing, or culture”.^4^ Of these options, clinical response to broad-spectrum antimicrobial agents, the so-called trial of antibiotics, has long been the priority for national programmes in resource-limited settings (appendix p 4).

The trial of antibiotics serves two distinct goals: first, to empirically treat bacterial respiratory tract infections using one or more antibiotics with minimal or no anti-mycobacteriological activity; and second, to use the response to treatment to determine the need for further tuberculosis investigations, assuming that illness due to active tuberculosis will not respond. The focus of this systematic review is on the second diagnostic goal, whereby a trial of antibiotics is used to distinguish tuberculosis from other infectious causes of respiratory illness.

We estimated conservatively that at least 26·5 million courses of antibiotics are prescribed in the course of diagnosing 5·3 million smear-negative tuberculosis registrations per year, which raises concerns about the contributions of this practice to antimicrobial resistance.^8^ This estimate assumes an average of five antibiotic courses per treatment initiation for a sputum-negative patient, including two courses given to the patient before tuberculosis treatment and three more given when tuberculosis is ruled out by the patient's response to antibiotics.^5^, ^7^ Despite the widespread use of the trial of antibiotics, no systematic review has focused on its diagnostic performance.

**Research in context**

**Evidence before this study**

Antimicrobial resistance and tuberculosis are both serious threats that together cause 2·5 million deaths each year, are part of the 2030 agenda for sustainable development, and are two of only five health issues to ever secure a dedicated United Nations High Level Meeting. Apart from drug-resistant tuberculosis, a less discussed but key overlap between these two threats is that tens of millions of doses of broad-spectrum antibiotics are used in the diagnostic work-up for tuberculosis, with the so-called trial of antibiotics probably being the most used tuberculosis diagnostic globally. The trial of antibiotics reflects the suboptimal nature of current tuberculosis diagnostics, which miss a substantial fraction of tuberculosis cases. The underlying assumptions are that symptoms that respond to antibiotics are attributable to other respiratory infections (assumed to be sensitive to the broad-spectrum antibiotic used), whereas non-responsive symptoms are likely to be due to tuberculosis.

Two previous systematic reviews documented the role of broad-spectrum antibiotics in the diagnosis of tuberculosis, although neither addressed their specific diagnostic value. The scarcity of evidence in this area was first highlighted in the 2007 WHO guidelines on tuberculosis diagnosis in HIV-prevelant and low-resource settings, which recommended the use of antibiotics in patients with HIV to treat presumptive bacterial infections, but not for diagnostic purposes. The 2018 WHO recommendations, however, retain response to antibiotic treatment as a key part of clinical evaluation of patients both with and without HIV following a negative Xpert MTB/Rif test.

**Added value of this study**

To our knowledge, this is the first systematic review and meta-analysis, and the most comprehensive assessment, of the performance of the trial of antibiotics in tuberculosis diagnostic algorithms. Our study shows little evidence to support the continued implementation of the trial of antibiotics. The available studies are few in number, of poor quality, and do not use standardised methodologies, leading to high interstudy heterogeneity. The pooled sensitivity (67%, 95% CI 42–85; *I*^2^=96%) and specificity (73%, 58–85; *I*^2^=99%) of the trial of antibiotics versus sputum mycobacteriology were both below internationally defined minimum performance profiles for tuberculosis diagnostics.

**Implications of all the available evidence**

The trial of antibiotics, despite being part of global recommendations for over three decades, has yet to be supported by evidence. The poor diagnostic performance, potential to increase antimicrobial resistance, and public health consequences of the misclassification of tuberculosis status warrant urgent and well designed prospective trials.

Other important evidence gaps concern the choice of antibiotics for the trial of antibiotics (except for the advice to avoid those with known anti-tuberculosis activity), the duration of treatment, the number of antibiotic trials, and the definition of treatment response. The inadequate consolidation of evidence in these areas is reflected in pronounced variations in how the trial of antibiotics is implemented across national programmes.^5^

The poor evidence on the use of the trial of antibiotics is also reflected in WHO recommendations, which evolved from bold recommendation of a routine trial of antibiotics in 1997^3^ to more cautious language in 2018.^4^ The 1997 WHO guidelines^3^ included the absence of a clinical response after 1 week of broad-spectrum antibiotics as part of the case definition for smear-negative tuberculosis. 10 years later, in 2007, the guidelines for people living with HIV or AIDS called for more research into the diagnostic benefit of the trial of antibiotics and recommended that the primary role of antibiotics should not be as a diagnostic aid but as treatment for concomitant bacterial infection.^9^ After another decade, and in the context of growing concern about antimicrobial resistance, the 2018 WHO model algorithms still support the trial of antibiotics (appendix p 3).^4^ In practice, national guidelines and routine clinical practice in low-income settings still follow the 1997 approach to the trial of antibiotics (appendix p 4).

The objective of this systematic review was to assess existing evidence for the diagnostic accuracy (sensitivity and specificity) of the trial of antibiotics compared with sputum mycobacteriology tests for the diagnosis of tuberculosis. We also describe the choice of antibiotic, duration of treatment, and definition of post-treatment improvement.

## Methods

### Search strategy and selection criteria

For this systematic review and meta-analysis, we searched MEDLINE, Embase, and Global Health using the Ovid platform for studies published up to March 15, 2019, when the search was run. The search strategies are described in the appendix (pp 1–2). We included all studies published in any language that included adults (≥15 years) who were being investigated for pulmonary tuberculosis, which reported outcomes of both a trial of antibiotics and mycobacteriology investigations as part of a standardised diagnostic work-up. Acceptable study designs were cross-sectional, cohort, or randomised controlled trials. To be eligible, studies had to recruit adults on the basis of symptoms suggestive of tuberculosis (with or without a preceding chest radiograph), include a trial of antibiotics as the index test and any sputum-based mycobacteriology test as the reference test, and report the proportions of participants whose mycobacteriology tests were positive or negative who were correctly or incorrectly identified through a trial of antibiotics (ie, both sensitivity and specificity).

The protocol for this systematic review, including detailed methods, is published elsewhere.^10^ This study is registered with the International Prospective Register of Systematic Reviews (PROSPERO), registration number CRD42017083915. We prepared our study protocol, performed the systematic review, and prepared the report according to recommendations by the Preferred Reporting Items for Systematic Reviews and Meta-Analyses (PRISMA).^11^

### Data extraction

Two reviewers independently screened the titles and abstracts of the articles identified through the electronic searches against the eligibility criteria: THD and MN assessed articles published from Jan 1, 1993, to March 15, 2019; and on Aug 6, 2019, following the advice of a peer reviewer, THD and CK assessed all articles indexed by the selected databases up to Dec 31, 1992. THD, MN, and CK independently assessed the full texts of the included papers, documented the reasons for non-inclusion, and identified additional articles from reference lists. KLF resolved disagreements in eligibility. Huan Zhang and Mengyun Liu (London School of Hygiene & Tropical Medicine, London, UK) independently assessed the full texts of Chinese-language articles. THD, MN, and CK extracted data from the eligible articles into an Excel database and resolved discrepancies by consensus.

The following data were extracted from eligible papers: first author, year of publication, country of data collection, antibiotics used for the trial of antibiotics, duration of antibiotic treatment, method of assessing response to antibiotic treatment, reference mycobacteriology tests, and number of patients given both a trial of antibiotics and a mycobacteriology reference test. Articles were defined as eligible for meta-analysis estimation of sensitivity and specificity if they provided data on numbers of patients that were true positives, false positives, false negatives, and true negatives. For studies with missing or incomplete information for the meta-analysis, we contacted the authors for data. In cases where data were unavailable, we included in narrative synthesis as much information as the study could provide.

### Assessment of study bias

We assessed risk of bias at the level of the study using QUADAS-2 (University of Bristol, Bristol, UK), the recommended tool for evaluating primary studies for inclusion in systematic reviews involving assessment of diagnostic accuracy.^12^ We assessed the risk of bias and applicability concerns using four domains: patient selection, index test, reference standard, and patient flow and timing of tests. The level of risk or concern was reported as either high, low, or unclear.

### Meta-analysis

We included in the meta-analysis all studies that provided data that allowed us to calculate sensitivity and specificity of a trial of antibiotics against a reference standard of mycobacteriology tests. The meta-analysis was done using MIDAS (version 15.0),^13^ which uses joint modelling of sensitivity and specificity. We estimated point estimates and 95% CIs for sensitivity and specificity for each study and for pooled data using bivariate random effects modelling.

To provide an inference of diagnostic quality, we plotted a summary receiver operating characteristic curve, in which the diagnostic accuracy of the trial of antibiotics was estimated by the area under the curve and the summary operating point.

We assessed heterogeneity across studies using the *I*^2^ statistic, and we used a bagplot to examine the spread of the observed data and identify outliers. We examined clinical utility of trial of antibiotics using a Fagan plot, and we used the Deeks funnel plot to identify evidence of publication bias in studies of diagnostic performance.

We did subgroup and sensitivity analyses. For the subgroup analysis, we used univariate meta-regression. Our a-priori subgroups were study setting (whether a study was done in sub-Saharan Africa) and reference test (whether the study used MTB culture as the reference standard). In a post-hoc analysis, we stratified the data by use of chest radiography (in addition to tuberculosis symptoms) for pre-screening. For the sensitivity analyses, we restricted the meta-analysis to high-quality studies (showing high risk of bias in no more than one domain of QUADAS-2).

### Role of the funding source

The funders had no role in the study design, data collection, data analysis, data interpretation, or in the writing of the manuscript. The corresponding author had full access to all the data in the study and had final responsibility for the decision to submit for publication.

## Results

**Figure 1:**
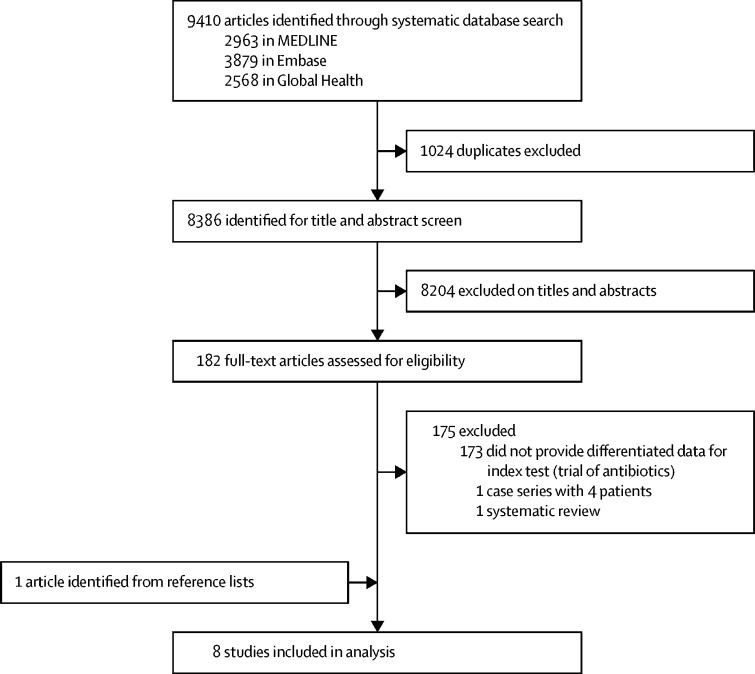
Study selection

We identified 9410 articles from the electronic searches, which reduced to 8386 after removing duplicates and to 182 after screening of the title and abstract (figure 1). After a full-text review, seven articles were included in the systematic review, which increased to eight following review of reference lists (figure 1).

The eight eligible studies were published between 1997 and 2016 and included 2786 participants from seven countries in Africa,^7^, ^14^, ^15^, ^16^, ^17^ South America,^18^ and Asia^19^, ^20^ (table). Seven studies evaluated participants in hospital settings or in clinics specialised in care of patients with HIV and tuberculosis. Two studies recruited only participants who were HIV-positive and one was restricted to participants who were HIV-negative. In all studies, the trial of antibiotics was used in a pre-screened population who tested tuberculosis-negative by smear microscopy. In addition to microscopy, three studies required a chest radiograph, but each of these excluded patients on the basis of a different radiographical finding: either features that were consistent with acute pneumonia,^17^ suggestive of respiratory diseases other than tuberculosis or other pathologies such as cardiac disorders,^7^ or suggestive of tuberculosis.^14^ Six studies used MTB culture as their reference diagnostic test, with samples collected from smear-negative participants at baseline, before antibiotics were prescribed. The remaining two studies^15^, ^20^ first prescribed antibiotics to smear-negative participants at baseline and then collected sputum for a combination of MTB culture and smear microscopy (the reference standard) on the same day as evaluation for treatment response (index test outcome).

**Table:** Characteristics of included studies

	**Setting**	**Study design**	**Proportion HIV positive**	Screened population*	Pre-screening assessments*	**Reference diagnostic test**	**Antibiotic, dose, and duration**	**Follow-up (days from baseline)**	**Definition of clinical response**	**Contribution to review population (n=2786)**
Wilkinson et al (1997)^17^	South Africa; hospital inpatients	Cohort	58% of target population, but study-specific proportion not reported	≥3 weeks' cough and sputum production, weight loss, night sweats, or chest pain	Three negative smears and chest x-ray; patient excluded if clinical and radiological features of acute pneumonia were present	MTB culture (Lowenstein–Jensen and Middlebrook 7H11 agar)	Ampicillin 500 mg four times daily for 7–10 days	Not reported	Not reported	237 (9%)
Wilkinson et al (2000)^7^	South Africa; hospital inpatients	Cohort	70%	≥3 weeks' respiratory symptoms (cough, chest pain, sputum production, shortness of breath, tachypnoea, or haemoptysis) and an abnormal chest x-ray compatible with tuberculosis; or community-acquired pneumonia (acute cough, fever, and sputum production) that did not respond to antibiotic treatment taken as an outpatient	Three negative smears and chest x-ray; patient excluded if clinical and radiological features consistent with other respiratory infections or cardiac pathologies were present	MTB culture (Lowenstein–Jensen and Middlebrook 7Hll agar)	Amoxycillin 500 mg three times daily for 5 days (erythromycin 500 mg four times daily given if no improvement from amoxycillin)	5	Patients met all four criteria: (1) cough ceased or substantially decreased (reported by both nurse and patient); (2) sputum production ceased or substantially decreased (measured in sputum container); (3) apyrexial for 48 h (measured on temperature chart); and (4) judgment by attending clinician, including above and change in pulse and respiratory rates	120 (4%)
Kudjawu et al (2006)^15^	Guinea; primary care clinic	Cohort	15%	≥3 weeks' cough; patient excluded if they were previously diagnosed with chronic lung disease, had received more than 72 h treatment for the acute condition that prompted consultation, or had a history of tuberculosis	Three negative smears	Smear microscopy (Ziehl–Neelsen and phenolauramine) or MTB culture (culture type not reported)	Amoxicillin 1500 mg daily for 10 days	14	Clinical definition: diminished cough, defervescence, and improved wellbeing; radiographical definition: appreciable clearing on day 14 film of densities noted on day 1 film	359 (13%)
Siddiqi at al (2006)^20^	Pakistan; tuberculosis clinic at referral hospital	Cohort	Not reported	≥3 weeks' cough; patient excluded if they had a history of tuberculosis or were on anti-tuberculosis therapy	Three negative smears	Smear microscopy or MTB culture (culture type not reported)	Penicillin or macrolide (dose not reported) for 7–10 days	7 to 10	Clinical judgment of a study-trained physician (no specific definition provided)	1000 (36%)
Soto et al (2011)^18^	Peru; referral hospital (26% inpatients; 74% referred from peripheral centres)	Cohort	0%	≥2 weeks' cough plus at least one of dyspnoea, thoracic pain, fever, night sweating, or weight loss	Three negative smears	MTB culture (Ogawa, Middlebrook 7H9 media, and mycobacteria growth indicator tube)	Doxycycline 100 mg twice daily for 10 days	14	Reduction or resolution of constitutional and respiratory symptoms plus resolution of signs at clinical examination	264 (9%)
Huerga et al (2012)^14^	Kenya, tuberculosis clinic at referral hospital	Cohort	68%	≥2 weeks' cough; patient excluded if they had taken fluoroquinolones or anti-tuberculosis drugs in the past month	Two negative smears and chest x-ray; patient excluded if chest x-ray suggested tuberculosis or if patient was in severe clinical condition	MTB culture (Lowenstein–Jensen and thin layer agar)	Amoxicillin 1 g three times daily for 5 days	5	Resolution judged as either complete resolution (resolution of all clinical symptoms with a normal physical examination), partial resolution (improvement with persistence of clinical symptoms or signs), or no resolution (absence of improvement or clinical worsening)	285 (10%)
Padmapriyadarsini et al (2013)^19^	India; network of HIV clinics	Cohort	100%	≥2 weeks' cough or fever in the past ≥2 weeks, or both	Three negative smears	MTB culture (Lowenstein–Jensen)	Amoxicillin 500 mg every 6 h for 7 days, followed by doxycycline 100 mg twice daily for 7 days	14	Patients considered not to have tuberculosis if they met all three criteria: (1) none or improved symptoms (cough or fever), (2) normal chest x-ray, and (3) negative sputum smears after 14 days	440 (16%)
Walusimbi et al (2016)^16^	Uganda; HIV clinic	Cohort	100%	≥2 weeks' cough or fever, or noticeable weight loss or excessive night sweats; patient excluded if they were on quinolone medication	Two negative fluorescent tuberculosis microscopy tests, and negative GeneXpert	MTB culture (mycobacteria growth indicator tube)	Macrolides and cephalosporins (dose and duration not reported)	14	Self-reported absence of symptoms to clinical staff	81 (3%)

MTB=*Mycobacterium tuberculosis*.

* Screened population refers to the eligibility criteria for the part of the study in which the index test was evaluated; pre-screening tests were done for eligible patients before the index test.

The choice of antibiotics for the trial of antibiotics varied across the studies, and four studies used more than one type of antibiotic. The most common class in the eight studies was penicillin, reported in six of the eight studies (table). Other antibiotic classes included macrolides in three studies, tetracyclines in two, and cephalosporins in one. The duration of treatment was also variable, ranging from 5 days to 14 days. Participants were assessed for their response to antibiotic treatment between 5 days and 14 days from the start of treatment. Although most studies implemented a single course of antibiotic treatment, two of them used two courses. One of these studies involved assessing the response to treatment before prescribing the second course,^7^ whereas the other study asked participants to return for assessment only after completing both courses.^19^

There was no consistent definition of the response to treatment, and approaches ranged from using self-reported improvement to using a combination of clinical and radiological assessments (table). The approaches for measuring the response to treatment were largely subjective in all studies. One study included in their definition for the outcome “a negative smear on day 14”.^19^ The treatment response evaluation approaches were more rigorous in studies involving hospitalised participants. For example, Wilkinson and colleagues^7^ used changes in cough, the amount of sputum produced, and body temperature as reported by a nurse. One study did not report how response to treatment was assessed.^17^

**Figure 2:**
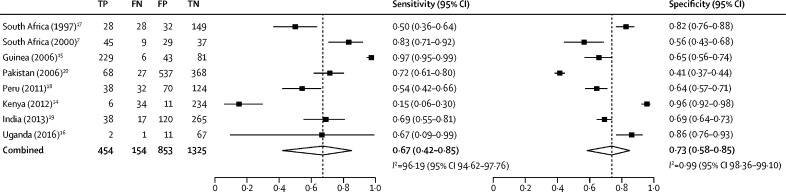
Diagnostic sensitivity and specificity of the trial of antibiotics versus mycobacteriology tests Meta-analysis of the diagnosis of pulmonary tuberculosis in the eight studies included. Mycobacteriology tests included culture only or culture plus smear microscopy. Dashed vertical lines show the pooled estimates. TP=true positive. FN=false negative. FP=false positive. TN=true negative.

**Figure 3:**
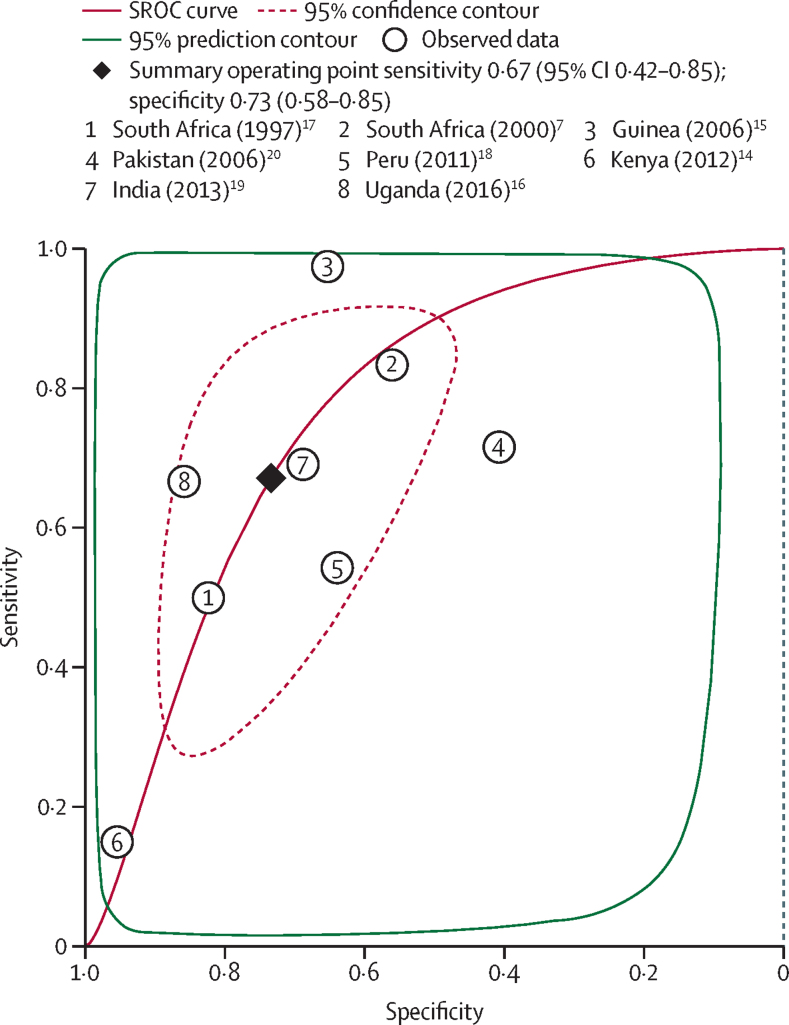
SROC meta-analysis of the diagnostic performance of the trial of antibiotics against reference mycobacteriology tests for diagnosing pulmonary tuberculosis in eight studies Area under the SROC is 0·77 (95% CI 0·73–0·80). Mycobacteriology tests included culture only or culture plus smear microscopy. The confidence contour shows the range that is likely to contain the population summary operating point and the prediction interval is the range that is likely to contain where study data that are not yet observed would fall. SROC=summary receiver operating characteristic curve.

All eight studies had disaggregated data, which allowed estimation of the pooled sensitivity and specificity of the trial of antibiotics compared with a mycobacteriology reference (MTB culture, smear microscopy, or both). The unadjusted individual study estimates for both specificity and sensitivity were not consistent across the studies (figure 2). Point estimates for sensitivity in the eight studies ranged from 15% to 97% (sample size range three to 235; median 56) and for specificity ranged from 41% to 96% (sample size range 66 to 905; median 188). Compared with mycobacteriology tests, the pooled sensitivity of the trial of antibiotics was 67% (95% CI 42–85; *I*^2^=96%) and the pooled specificity was 73% (58–85; *I*^2^=99%). The area under the summary receiver operating characteristic curve was 0·77 (95% CI 0·73–0·80; figure 3).

In subgroup analyses, pooled estimates of sensitivity and specificity by study setting and reference standard definition still showed substantial heterogeneity (appendix p 12), although these analyses should be interpreted with caution because of the small number of included studies. Sensitivity was lower and specificity higher in studies that used MTB culture alone for the reference standard, although again these need to be interpreted with caution considering the small numbers. Fagan's nomogram showed that if the prevalence of pulmonary tuberculosis is 20%, the trial of antibiotics would increase the probability of correctly detecting mycobacteriology-positive pulmonary tuberculosis in the study population by an absolute value of 19% (from a pre-test probability of 20% to a post-test probability of 39%). When participants reported resolution of symptoms after a course of antibiotics (ie, testing tuberculosis-negative for the trial of antibiotics), the probability that they could nonetheless have mycobacteriology-positive pulmonary tuberculosis was 10% (appendix p 5). The Deeks' funnel plot for the eight studies included in our meta-analysis indicated that there was no evidence of publication bias (p=0·84 for Deeks' funnel plot asymmetry test; appendix p 11).

Studies within the 95% confidence bounds of the median distribution in the bagplot were not clustered together (appendix p 6). There were two outliers (the 2006 study in Guinea^15^ and the 2012 study in Kenya),^14^ but excluding these studies from the meta-analysis in a post-hoc sensitivity analysis did not account for the substantial heterogeneity of the full model (appendix p 12). Of note, the 2012 Kenya study categorised outcomes of antibiotic treatment as either complete resolution, partial resolution, or no resolution (table) and considered only complete (not partial) resolution as improvement. However, to be consistent with the definitions used in the other eligible studies, we re-categorised the data from the 2012 Kenya study such that clinical improvement referred to any improvement (either partial or complete resolution) and no improvement referred to no resolution. Using the authors' definitions did not significantly change the pooled estimates for sensitivity and specificity.

Evaluating our main question (of the diagnostic accuracy of the trial of antibiotics compared with sputum mycobacteriology tests for the diagnosis of tuberculosis) against the eight studies, we established that each study had a potential risk of bias in at least one of the four domains of the QUADAS-2 tool (appendix pp 7–10). A sensitivity analysis that involved doing the meta-analysis without the one study that showed a high risk of bias in at least three QUADAS-2 domains yielded sensitivity, specificity, and *I*^2^ estimates that were similar to the full analysis (appendix p 12). In all studies, the patient selection process and conduct of both index and reference tests matched the expectation of our main question.

## Discussion

We report, to our knowledge, the first systematic review to assess rigorously the diagnostic performance of the trial of antibiotics against mycobacteriology for sputum-negative tuberculosis. Our main findings are that the available evidence base is insufficient and limited by incomplete geographical coverage and inconsistencies on the choice of antibiotics, duration of treatment, and case definition for post-treatment clinical improvement. However, the pooled sensitivity (67%) and specificity (73%) estimates fall well below minimum recommendations for new tuberculosis triage and diagnostic tests for adults.^21^ As the medical community moves towards meeting End TB goals,^22^ clinicians and those designing public health programmes need to be aware of how substantial the misclassification by trial of antibiotics can be.

Our results call for reconsideration of the appropriateness of retaining routine trial of antibiotics in any international guidelines and national tuberculosis diagnostic algorithms. Algorithms that instead promote mycobacteriology and early chest radiography, repeated as needed, are likely to have better diagnostic accuracy.^23^ Broad-spectrum antibiotics will still be needed to treat clinically suspected bacterial infection, with the crucial evidence gap then being how different antibiotic strategies affect clinical outcomes^24^ and antimicrobial resistance^25^ during tuberculosis investigation, including among key subgroups such as inpatients, people living with HIV, children, and participants identified through tuberculosis screening initiatives.

We identified only eight published studies investigating the diagnostic performance of the trial of antibiotics for tuberculosis, which is well below the number needed for making informed health-care choices. This number is especially striking given that tuberculosis is a life-threatening illness and that the trial of antibiotics might be the most commonly used tuberculosis diagnostic test globally,^26^ resulting in non-pathogen-directed prescription of tens of millions of doses of antibiotics each year.^27^ Consistent data from well performed randomised controlled trials are required for high-quality evidence,^28^ but our review did not identify any randomised controlled trials, and most of the observational studies that we identified were not optimally designed or sized. Instead, four of the eight studies included in this Article assessed the diagnostic performance of the trial of antibiotics as a secondary or exploratory outcome using a small subset of the original study population, reducing power and increasing the risk of selection bias. Methodological concerns are highlighted by the suboptimal scores for each included study on the QUADAS-2 tool for assessing risk of bias. The thin evidence and poor methodological quality that we have observed with the trial of antibiotics does not match the past 10 years' rapid accumulation of high-quality trial data informing the rational use of antibiotics for the treatment of presumed chest infections when tuberculosis is not under consideration.^29^

The poor diagnostic performance reported here is unsurprising given the wide differential of tuberculosis symptoms, including viral and non-infectious causes.^30^ Misleading responses could also arise from partial response to antibiotics in patients with tuberculosis with concurrent bacterial infections. This situation is best described in (but not limited to) patients with HIV, which led to the 2007 WHO recommendation to separately investigate and manage tuberculosis and bacterial infections in people living with HIV.^9^ Misclassifying tuberculosis is costly to both the patient and the health system. False-positive tuberculosis diagnoses expose patients to unnecessary tuberculosis chemotherapy and its associated toxicity, stigma, hospital visits, lost schooling or employment, and any consequences from delayed diagnosis of the true cause of illness. False-negative tuberculosis diagnoses are associated with the individual and public health consequences of delayed diagnosis and ongoing transmission.^31^

A framework for evaluating the diagnostic performance of the trial of antibiotics is provided by comparing our estimates against target product profiles for new non-sputum tuberculosis triage tests (minimum sensitivity of 90% and specificity of 70%) and sputum-based replacements for smear microscopy at the primary care level (minimum sensitivity of 60% for smear-negative tuberculosis and specificity of 98%).^21^ Additional attributes of diagnostic tests that are important to patients and are not met by the trial of antibiotics include timely diagnosis^5^, ^7^ and low cost (the recommendation from WHO^21^ of <US$6 for a new diagnostic will be exceeded with the trial of antibiotics once expenses incurred by patients,^32^ costs of drugs, and staff time^33^ are included). The main attributes that are likely to drive the continued use of the trial of antibiotics globally are, therefore, the ease with which prescription fits into the high throughput of consulting rooms, as well as patients' expectations and clinicians' habitual prescription of antibiotics for respiratory consultations—considerations that should be discouraged and not encouraged in an era of rising threat from antimicrobial resistance.^34^, ^35^, ^36^

Our meta-analysis showed substantial heterogeneity, which is consistent with the non-standardised nature of choice and duration of antibiotics and the definition of response to treatment. Other variables potentially affecting heterogeneity include site-specific factors, such as antibiotic resistance patterns and exposure to tobacco smoke and air pollution, level of health care, pre-study investigations (eg, whether chest radiography was done), and HIV prevalence. The small number of eligible studies limited our power to explore these variables. Altogether, the heterogeneity in and underlying differences between studies highlight the variations that exist in the interpretation of WHO guidelines in different settings.

The main limitations of this systematic review and meta-analysis are the small number of studies identified, the suboptimal number of participants per study, the pronounced variation in the definitions and methods used, and the suboptimal reference standard. Suboptimal reference standards are a concern for studies in tuberculosis diagnostics.^37^ The studies included in our review used either one or a combination of MTB culture and smear microscopy, each of which can misclassify patients' tuberculosis disease status, thereby misinterpreting the true sensitivity and specificity of the trial of antibiotics. We were unable to explore the probable causes of heterogeneity given the data limitations. We restricted our search strategy to peer-reviewed articles and will therefore have omitted eligible studies published in conference proceedings or in programme reports. We might also have missed some articles, including peer-reviewed papers, because data on trials of antibiotics are often reported as secondary or exploratory outcomes to the main study objective. In addition, the result of the Deeks' funnel plot should be interpreted with the understanding that the model works best if it has at least ten studies. In the absence of a better tool, we thought that Deeks' funnel plot could still give a reasonable estimate for publication bias.

The End TB Strategy calls for major expansion of tuberculosis testing to find the missing millions of undiagnosed tuberculosis cases and to save lives; making treatment available to the target of 40 million tuberculosis cases by 2022 will involve testing up to 1 billion people. The ethical obligation to minimise individual harms is especially pertinent in the context of systematic screening strategies, in which patients have not initiated the diagnostic process.^38^ Studies investigating the role, if any, of the trial of antibiotics in patients identified through systematic screening are missing from this meta-analysis but are urgently needed, both to minimise individual harms and from the equally important perspective of antibiotic stewardship.

In conclusion, despite more than 30 years of international guidelines and national algorithms promoting the trial of antibiotics for tuberculosis diagnosis, the small amount of data presented here on its diagnostic utility do not support the underlying rationale. Antibiotics might still be indicated for the treatment of suspected bacterial infections, but in line with strategies for addressing antimicrobial resistance, their use during tuberculosis investigations should otherwise be minimised. More data are needed to guide the minimisation of antibiotic use as we scale up tuberculosis testing globally. We urge donors to prioritise support for well conducted implementation research studies and randomised controlled trials that aim to evaluate rigorously the effect of different antibiotic strategies on outcomes such as short-term mortality, need for hospitalisation or so-called rescue antibiotics, and antimicrobial resistance. These studies should include trials of the safety of antibiotic minimisation protocols^29^ to support the rapid generation of sufficient data to guide evidence-based, patient-centred management of presumptive tuberculosis patients, including key subgroups and populations for whom the relative benefits and harms of antibiotics are likely to vary from routine clinic adults—notably, young children, people with HIV,^39^ people with diabetes, and tuberculosis screening participants.
