# Supplementary material for: Utility of broad-spectrum antibiotics for diagnosing pulmonary tuberculosis in adults: a systematic review and meta-analysis
Source: Lancet Infect Dis. 2020 Sep;20(9):1089–98. doi: 10.1016/S1473-3099(20)30143-2 (PMC7456780; doi:10.1016/S1473-3099(20)30143-2)
Supplement: Supplementary appendix [file mmc1.pdf]

THE LANCET Infectious Diseases

Supplementary webappendix

This webappendix formed part of the original submission and has been peer reviewed. We post it as supplied by the authors.

Supplement to: Divala TH, Fielding KL, Kandulu C, et al. Utility of broad-spectrum antibiotics for diagnosing pulmonary tuberculosis in adults: a systematic review and meta-analysis. *Lancet Infect Dis* 2020; published online May 18. https://doi.org/10.1016/S1473-3099(20)30143-2.

Utility of broad-spectrum antibiotics for diagnosing pulmonary tuberculosis in adults: a systematic review and meta-analysis

Appendix 1: Additional figures and tables

Appendix Table 1: Search strategy for MEDLINE using Ovid platform

Search line	Search terms
Part 1	*Defining study population:*
1.	exp Tuberculosis/
2.	tuberculosis.mp.
3.	(suspect* adj3 (TB or Tuberculosis)).mp.
4.	(presumpt* adj3 (TB or Tuberculosis)).mp.
5.	(probabl* adj3 (TB or Tuberculosis)).mp.
6.	exp Cough/
7.	tb.mp.
8.	(suspect* adj3 (TB or Tuberculosis)).mp.
9.	or/1-8
Part 2	*Defining study intervention*
10.	(Antibiotic* adj3 trial).mp.
11.	antibiotic*.mp.
12.	Anti-Bacterial Agents/
13.	(oral* adj3 antibiotic*).mp.
14.	(amox?cillin or erythromycin or azithromycin or doxycyclin* or Vibramycin or clavulanic acid or co-amoxiclav).mp.
15.	or/10-14
Part 3	*Defining study outcome*
16.	exp "Sensitivity and Specificity"/
17.	sensitivity.mp.
18.	specificity.mp.
19.	accuracy.mp.
20.	exp "Predictive Value of Tests"/
21.	((positive or negative) adj2 predictive value).mp.
22.	(ppv or npv).mp.
23.	or/16-22

Part 4	*Subject combinations*
24.	9 and 15 (*population and intervention*)
25.	23 and 24 (*Population and intervention and outcome*)
Part 5	*Applying pre-defined limits*
26.	limit 25 to yr="1993 -Current"

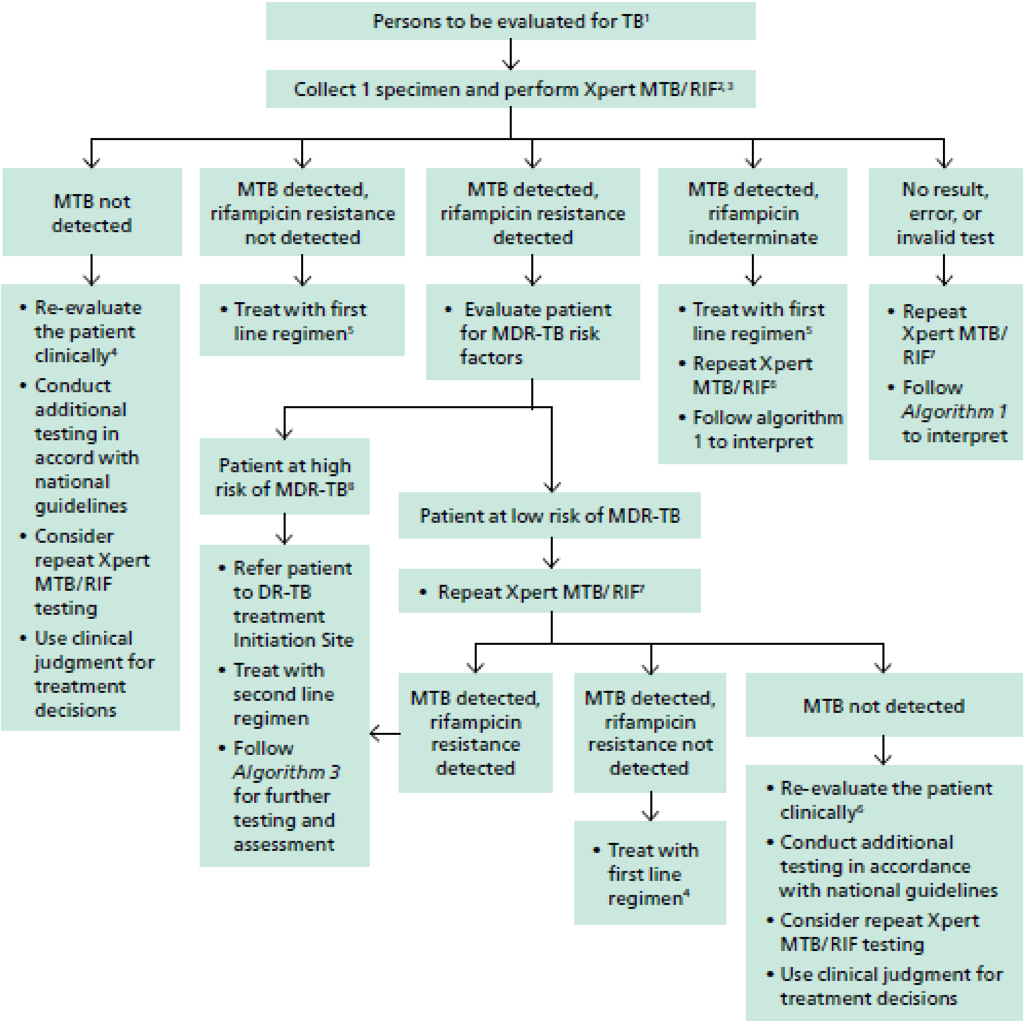

1. Persons to be evaluated for TB include adults and children with signs or symptoms suggestive of TB or with a chest X-ray with abnormalities suggestive of TB. This algorithm may also be followed for the detection of MTB using CSF, lymph node and other tissue specimen from persons being evaluated for extrapulmonary TB. For persons being evaluated for TB who are HIV positive and have CD4 counts ≤100 cells/μl or are seriously ill, see Algorithm 4.
2. The new generation Xpert MTB/RIF Ultra assay (Ultra) uses the same semi-quantitative categories used in the Xpert MTB/RIF assay, with an additional semi-quantitative category "trace call" that corresponds to the lowest bacillary burden for Mycobacterium tuberculosis (MTB) complex detection. If MTB is detected with a "trace call", then no interpretation can be made regarding rifampicin resistance and results should be reported as MTB detected, trace, RIF indeterminate (Follow section on "MTB detected, rifampicin indeterminate" under Algorithm 1). The "trace call" positive result is sufficient to initiate therapy in those with known or suspected HIV infection, children and for patients with extrapulmonary samples. For other categories of patients repeating test may be considered with use of second Ultra test for clinical decisions and patients follow-up. (See GLI Planning for country transition to Xpert MTB/RIF Ultra Cartridges).
3. Programmes may consider collecting two specimens upfront. The first specimen should be promptly tested using the Xpert MTB/RIF test. The second specimen may be used for the additional testing described in this algorithm. For persons being evaluated for pulmonary TB, sputum is the preferred specimen.
4. Further investigations for TB may include chest X-ray, additional clinical assessments, clinical response following treatment with broad-spectrum antimicrobial agents, repeat Xpert MTB/RIF testing, or culture.
5. Patients should be initiated on a first-line regimen according to national guidelines. A sample may be sent for molecular or phenotypic DST for isoniazid , particularly if the patient has been previously treated with isoniazid or if there is a high prevalence of isoniazid resistance not associated with rifampicin resistance (i.e., isoniazid mono- or poly-resistance) in this setting or for DST for rifampicin if rifampicin resistance is still suspected.
6. Repeat Xpert MTB/RIF test at the same testing site with a fresh specimen. Use the rifampicin result of the second Xpert MTB/RIF test in this algorithm for a decision(s) regarding choice of regimen (first line or second line regimen).
7. Repeat Xpert MTB/RIF test at the same testing site with a fresh specimen. Interpret the result of the repeat test as shown in this algorithm. Use the result of the second Xpert MTB/RIF test for clinical decisions.
8. Patients at high risk for multidrug-resistant TB (MDR-TB) include previously treated patients including those who had been lost to follow-up, relapsed, and failed a treatment regimen; non-converters (smear positive at end of intensive phase); MDR-TB contacts; and any other MDR-TB risk groups identified in the country.

Appendix Figure 1a: WHO/GLI Model Algorithm 1; Preferred algorithm for universal patient access to rapid testing to detect MTB and rifampicin resistance (June 2018)

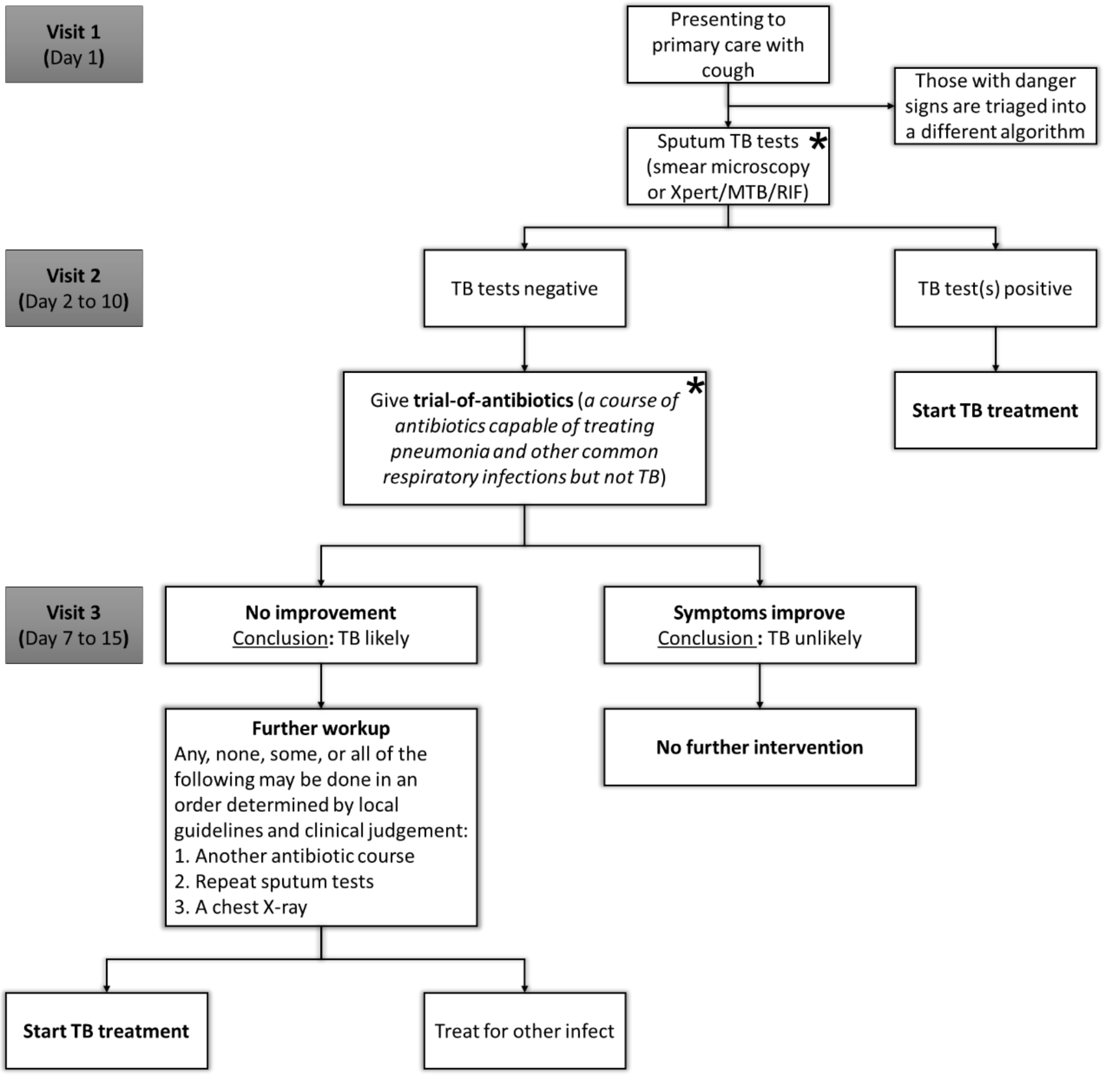

*The common clinical practice is that outpatients start antibiotics at the time of submitting sputum, to avoid the need for a third clinic visit to complete the algorithm. In some guidelines, trial-of-antibiotics is implemented after chest X-ray.

Appendix Figure 1b: The position of trial-of-antibiotics in most national tuberculosis diagnostic algorithms showing how countries interpret the WHO GLI model guidelines (based on national guidelines from Ghana, Malawi and South Africa).

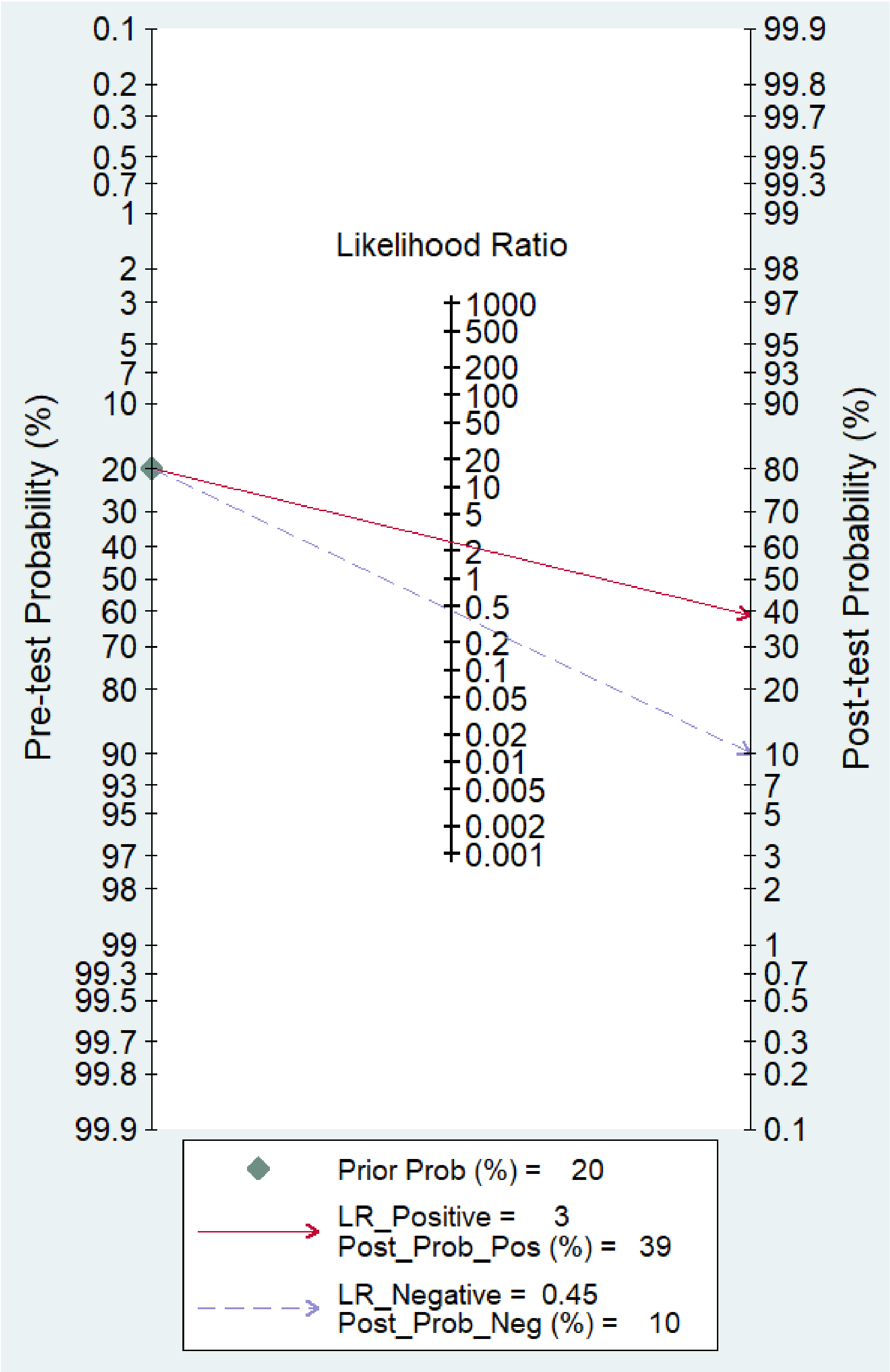

Appendix figure 2: Fagan's nomogram demonstrating clinical utility of trial-of-antibiotics by plotting post-test probabilities of detecting mycobacteriology positive PTB. In this analysis, the pre-test probability, fixed at 20%, is investigators suggestion of TB prevalence based on reference standard diagnosis. The interpretation of the post-test probabilities is as follows: with an estimated TB prevalence of 20%, if a patient tests positive using trial-of-antibiotics, the probability that they truly have TB is 39% (solid line in red); if patient tests negative, the probability that they have TB is 10% (blue dotted line).

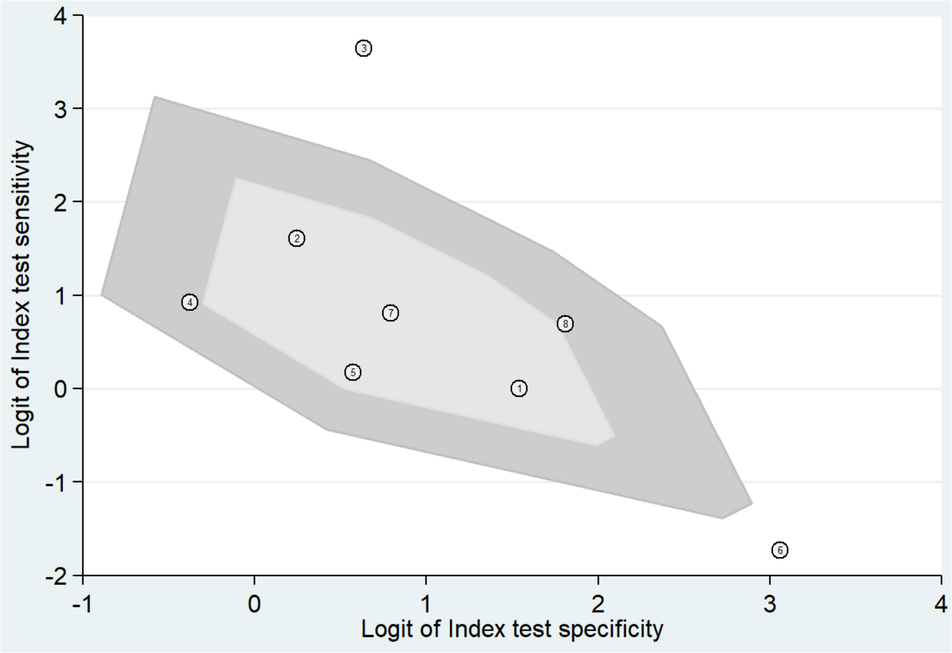

Study ID	country, year
1	South Africa, 1997
2	South Africa, 2000
3	Guinea, 2006
4	Pakistan, 2006
5	Peru, 2011
6	Kenya, 2012
7	India, 2013
8	Uganda, 2016

Appendix figure 3: The Bagplot demonstrating the level of heterogeneity using the spread of the 8 studies included in meta-analysis

Appendix table 3: Assessment of the quality of included studies against the review question using QUADAS 2 tool (University of Bristol)

Author	South Africa	South Africa	Guinea	Pakistan	Peru	Kenya	India	Uganda
Year	**1997**	**2000**	**2006**	**2006**	**2011**	**2012**	**2013**	**2016**
**Domain 1: Patient selection**								
Was a consecutive or random sample of patients enrolled?	yes	yes	yes	yes	yes	yes	yes	yes
Was a case-control design avoided?	yes	yes	yes	yes	yes	yes	yes	yes
Did the study avoid inappropriate exclusions?	No, one of their exclusion criteria was clinical picture consistent with pneumonia	No, they excluded patients based on clinical and radiological features consistent with pneumonia. Inclusion was also based on CXR consistent with TB.	yes	No, 64% of the 2794 patients treated with antibiotics did not have their outcome evaluated (loss to follow up)	yes	No, 66 of 380 patients were excluded from receiving antibiotics and put on presumptive TB treatment either based on CXR or other clinical TB diagnosis	No, started 17 patients on TB treatment based on clinical judgement and excluded them from receiving antibiotics	No, study started with 162 patients, 157 received antibiotics and reported outcome; but only 110 patients had culture done, of which only 81 had valid results
**Could the selection of patients have introduced bias? (Low if YES to all above; High if any NO)**	**high risk**	**high risk**	**low risk**	**high risk**	**low risk**	**high risk**	**high risk**	**high risk**

Author	South Africa	South Africa	Guinea	Pakistan	Peru	Kenya	India	Uganda
Year	**1997**	**2000**	**2006**	**2006**	**2011**	**2012**	**2013**	**2016**
**Is there concern that the included patients do not match the review question?**	**low risk**	**low risk**	**low risk**	**low risk**	**low risk**	**low risk**	**low risk**	**low risk**
**Domain 2: Index test**								
Were the results of trial of non-TB antibiotics interpreted without knowledge of the results of the reference standard?	Yes	Yes	Yes	Yes	Yes	Yes	Yes	Yes
Was the interpretation approach of trial-of-antibiotics outcome pre-specified?	No	Yes	Yes	No	Yes	Yes	No	Yes
**Could the conduct or interpretation of trial-of-antibiotics as a diagnostic test have introduced bias? (Low if YES to all above; High if any NO)**	**high risk**	**low risk**	**low risk**	**high risk**	**low risk**	**low risk**	**high risk**	**low risk**
**Is there concern that the trial of antibiotics, its conduct, or interpretation differ from the review question?**	**low risk**	**low risk**	**low risk**	**low risk**	**low risk**	**low risk**	**low risk**	**low risk**
**Domain 3: Reference test**								
Is the reference TB microbiology test likely to correctly detect TB?	Yes	Yes	Yes	Yes	Yes	Yes	Yes	Yes

Author	**South Africa**	**South Africa**	**Guinea**	**Pakistan**	**Peru**	**Kenya**	**India**	**Uganda**
Year	**1997**	**2000**	**2006**	**2006**	**2011**	**2012**	**2013**	**2016**
Were the TB microbiology test results interpreted without knowledge of the outcome of the trial of non-TB antibiotics?	Yes	Yes	Yes	Yes	Yes	Yes	Yes	Yes
**Could the TB microbiology test, its conduct, or its interpretation have introduced bias? (Low if YES to all above; High if any NO)**	**low risk**	**low risk**	**low risk**	**low risk**	**low risk**	**low risk**	**low risk**	**low risk**
**Is there concern that the target condition as defined by the reference standard in the paper does not match the review question?**	**low risk**	**low risk**	**low risk**	**low risk**	**low risk**	**low risk**	**low risk**	**low risk**
**Domain 4: Patient flow**								
Was there an appropriate interval between antibiotics and reference TB microbiology test?	Yes	Yes	No, sample for reference standard was taken while index test outcome was known	No, sample for reference standard was taken while index test outcome was known	Yes	Yes	Yes	Yes
Did all the included patients have a TB microbiology test?	Yes	Yes	Yes	Yes	Yes	Yes	Yes	No

Author	South Africa	South Africa	Guinea	Pakistan	Peru	Kenya	India	Uganda
Year	**1997**	**2000**	**2006**	**2006**	**2011**	**2012**	**2013**	**2016**
Did patients receive the same TB microbiology test?	Yes	Yes	No, used smear for reference in some patients, in those who were smear negative, used culture	Yes	Yes	Yes	Yes	Yes
Were all patients who received index test included in the analysis?	Yes	Yes	yes	No, 64% of patients were lost to follow up	No, 21 of 285 were lost to follow up of	No, final sample missing 32 patients due to inconclusive culture	yes	No, 76 of 167 patients with available index test outcome had no valid reference test results
**Could the patient flow have introduced bias? (Low if YES to all above; High if any NO)**	**low risk**	**low risk**	**high risk**	**high risk**	**high risk**	**high risk**	**low risk**	**high risk**

**Number of high-risk domains out of four**	**2**	**1**	**2**	**3**	**1**	**2**	**2**	**2**

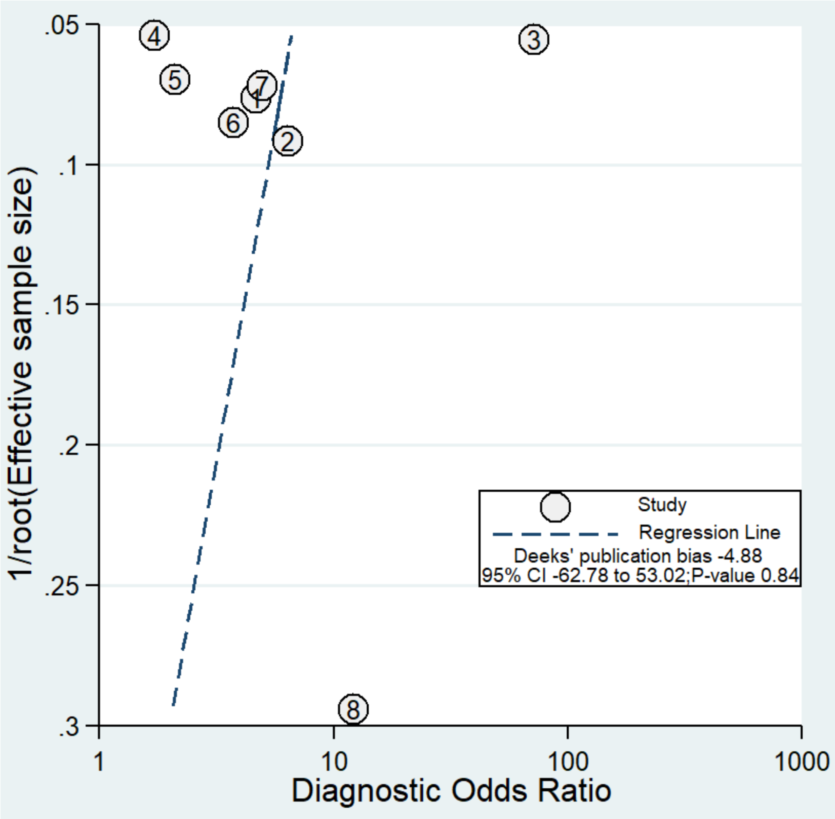

Study ID	country, year
1	South Africa, 1997
2	South Africa, 2000
3	Guinea, 2006
4	Pakistan, 2006
5	Peru, 2011
6	Kenya, 2012
7	India, 2013
8	Uganda, 2016

Appendix figure 4: Deeks' funnel plot to evaluate publication bias in the 8 studies included in the meta-analysis

Appendix table 2a: Subgroup analysis

Covariate (refer to Table 1)	category	Number of studies	Sensitivity (95% CI)	p-value for difference in sensitivity	Specificity (95% CI)	p-value for difference in specificity	Joint model I^2 (%)
Sub-Saharan Africa	Yes	5	0.69 (0.41, 0.97)	0.83	0.81 (0.70, 0.92)	0.35	72
	No	3	0.65 (0.29, 1.00)		0.58 (0.36, 0.80)		
Culture only for reference standard	Yes	6	0.55 (0.31, 0.79)	0.03	0.79 (0.68, 0.90)	0.24	52
	No	2	0.90 (0.76, 1.00)		0.53 (0.24, 0.81)		
These analyses are exploratory and should be interpreted with caution considering the small number of included studies.							

Appendix table 2b: Sensitivity analyses attempting to explain high heterogeneity

Description	Included studies	Sensitivity (95% CI)	Sensitivity I^2 (95% CI)	Specificity (95% CI)	Specificity I^2 (95% CI)
All studies	1, 2, 3, 4, 5, 6,7, and 8	67 (42, 85)	96 (95, 98)	73 (58, 85)	99 (98, 99)
Excluding studies (1/8) based on quality (with high risk of bias in at least three domains of Quadas 2 tool).	1,2, 3, 4, 5, and 7	66 (37, 87)	97 (95, 98)	77 (64, 87)	95 (92, 97)
Excluding studies (2/8) outside the 95% CI of the median distribution of the bagplot.	1, 2, 4, 5, 7, and 8	64 (53, 74)	82 (69, 96)	67 (53, 79)	98 (98, 99)
**1.** South Africa, 1997<br>**2.** South Africa, 2000<br>**3.** Guinea, 2006<br>**4.** Pakistan, 2006			**5.** Peru, 2011<br>**6.** Kenya, 2012<br>**7.** India, 2013<br>**8.** Uganda, 2016		
These analyses are exploratory and should be interpreted with caution considering the small number of included studies.					

Appendix 2: Stata Code for meta-analysis

A. Preliminary steps:
   i. Start stata as administrator
   ii. Install (if not installed) midas
   iii. Install (if not installed) metan
   iv. Install (if not installed) mylabels
   v. Install (if not installed) gllamm

B. Load the following data

Studyid	*author*	*year*	*sampsize*	*tp*	*fn*	*fp*	*tn*	*reference*	*ref*	*country*	*region-ssa*
1	South Africa	1997	237	28	28	32	149	culture	1	South Africa	1
2	South Africa	2000	120	45	9	29	37	culture	1	South Africa	1
3	Guinea	2006	359	229	6	43	81	Smear+culture	0	Guinea	1
4	Pakistan	2006	1000	68	27	537	368	Smear+culture	0	Pakistan	0
5	Peru	2011	264	38	32	70	124	culture	1	Peru	0
6	Kenya	2012	285	6	34	11	234	culture	1	Kenya	1
7	India	2013	440	38	17	120	265	culture	1	India	0
8	Uganda	2016	81	2	1	11	67	culture	1	Uganda	1

C. Perform the following analyses

*Summary Statistics

```
midas tp fp fn tn, res(all)
```

*Forest plot to demonstrate study-specific on right y-axis

```
midas tp fp fn tn, id(author year) ms(0.75) ford fors bfor(dss)
```

*Summary ROC Curve with prediction and confidence Contours

```
midas tp fp fn tn, plot sroc(both)
```

*Linear regression test of funnel plot asymmetry

```
midas tp fp fn tn, pubbias
```

*Fagan's plot

```
midas tp fp fn tn, fagan(0.20)
```

*Bagplot

```
midas tp fp fn tn, bivbox scheme(s2color)
```
